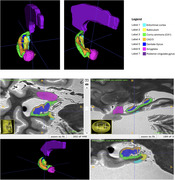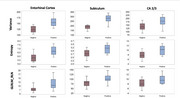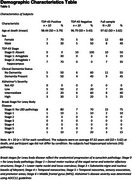# Radiomics Analysis of Hippocampal Subfields in Limbic‐Predominant Age‐related TDP‐43 Encephalopathy (LATE)

**DOI:** 10.1002/alz70861_108693

**Published:** 2025-12-23

**Authors:** Abhi P Jain, Harmony Cen, Bino Varghese, Davis C. Woodworth, Seyed Ahmad Sajjadi, Nasim Sheikh‐Bahaei

**Affiliations:** ^1^ University of Southern California, Los Angeles, CA USA; ^2^ Keck School of Medicine, University of Southern California, Los Angeles, CA USA; ^3^ University of California, Irvine, Irvine, CA USA; ^4^ Institute for Memory Impairments and Neurological Disorders (UCI‐MIND), University of California, Irvine, Irvine, CA USA

## Abstract

**Background:**

Limbic‐predominant age‐related TDP‐43 encephalopathy (LATE) is a prevalent neurodegenerative disorder in elderly individuals, characterized by clinical overlap with Alzheimer’s Disease (AD) but distinct neuropathological features, primarily TDP‐43 proteinopathy in limbic structures. Currently, the absence of definitive in vivo imaging biomarkers makes clinical differentiation of LATE from AD challenging.

**Method:**

We scanned formalin‐submerged hemispheres or whole brains donated by participants from the 90+ Study on a Siemens Prisma MRI scanner using high resolution structural sequences. We selected 10 participants with LATE and 10 participants without LATE neuropathology (Table 1), all without significant concomitant AD neuropathologic change or hippocampal sclerosis of aging. We performed manual segmentation (Figure 1) of hippocampal subfields (dentate gyrus, CA1, CA2/3, DG/CA4, subiculum, entorhinal cortex, and posterior cingulate gyrus) and amygdala using a 0.4mm isotropic T2w SPACE scan. We performed radiomic analysis of the Regions of Interests using the Cancer Imaging Phenomics Toolkit (CaPTk), extracting 129 radiomic metrics from six texture families: Intensity, Histogram, Gray‐Level Co‐occurrence Matrix (GLCM), Gray‐Level Run Length Matrix (GLRLM), Gray‐Level Size Zone Matrix (GLSZM), and Neighboring Gray Tone Difference Matrix (NGTDM). We compared values for these measures across groups using T‐tests.

**Result:**

Radiomic analysis revealed trends for greater variability of signal and textural features in participants with LATE, suggesting greater tissue heterogeneity: the entorhinal cortex, subiculum, and CA2/3 subfields. Specifically, increased variance, entropy, and GLRLM Run Length Non‐Uniformity (RLN) were noted, indicating greater tissue heterogeneity in LATE‐positive cases (Figure 2). Additionally, CA1 demonstrated higher NGTDM complexity and contrast (*p* =0.03 and *p* =0.04, respectively), suggesting increased textural complexity. The amygdala exhibited no statistically significant increases in standard deviation (*p* =0.07) and Median Absolute Deviation (MAD; *p* =0.06).

**Conclusion:**

Radiomic metrics, particularly those derived from the entorhinal cortex, subiculum, and CA2/3 regions, revealed distinct patterns of change that may enhance the diagnostic accuracy of LATE in vivo. While similar patterns were observed in the CA1 and amygdala regions, the changes were modest and did not reach statistical significance—likely due to limited sample size. Nevertheless, these radiomic features, if validated in larger cohorts, hold promise as non‐invasive biomarkers for improved clinical differentiation, diagnosis, and early management of LATE.